# A Rare Case of HHV-6 Encephalitis in an Immunocompetent Host: Case Report and Literature Review

**DOI:** 10.7759/cureus.23007

**Published:** 2022-03-09

**Authors:** Erinie Mekheal, Ariana R Tagliaferri, Kevin s Vasquez, Rovena Pjetergjoka, Gabrielle Lobue, David Townsend, Konstantinos Leou, Monisha Singhal

**Affiliations:** 1 Internal Medicine, St. Joseph's Regional Medical Center, Paterson, USA; 2 Family Medicine, St. Joseph's Regional Medical Center, Paterson, USA; 3 Internal Medicine, St. Joseph's Regional Medical Center, paterson, USA; 4 Internal Medicine, St George's University School of Medicine, Paterson, USA; 5 Critical Care Medicine, St. Joseph's Regional Medical Center, Paterson, USA

**Keywords:** temporal lobe epilepsy, immunocompetent adult, encephalitis, meningoencephalitis, human herpesvirus-6 (hhv-6)

## Abstract

Human herpesvirus-6 (HHV-6) is a virus known for causing the highly contagious infection, roseola infantum, and has been associated with causing encephalitis in pediatric patients and less commonly in adult patients as well. Regardless of the patient's age, the primary HHV-6 infection could be complicated by neurological sequelae including encephalitis, acute encephalopathy with biphasic seizures syndrome, or demyelinating disease. HHV-6 encephalitis does occur in an adult as a primary infection or reactivation. However, immunocompromised, hematopoietic stem cell transplantation patients, and solid organ transplant recipients are the most affected population. Here we present a rare case of HHV-6 encephalitis in a 26-year-old healthy immunocompetent male. HHV-6 viral DNA was detected in the cerebrospinal fluid during the acute stage of the disease, and the diagnosis was confirmed by quantitative polymerase chain reaction (PCR). The patient was treated with ganciclovir and had a complete response to treatment without any further complication. The pathophysiology, clinical course, and treatment in otherwise immunocompetent adult patients are also discussed.

## Introduction

Human herpesvirus-6 (HHV-6) is a double-stranded DNA virus of the Roseolovirus family. HHV-6 is more commonly known for exanthem subitum (roseola infantum or sixth disease). HHV-6 can remain in the latent state after primary infection with potential for future reactivation. In the adult population, especially in the immunocompromised, HHV-6 has been associated with encephalitis and meningoencephalitis, in addition to other disseminated diseases. Additional neurological manifestations have been reported, such as febrile seizures, meningitis, and epilepsy, in both immunocompromised and immunocompetent individuals. Studies showed that the adult population might be seropositive for HHV-6, without presenting any risk of active viral infection, known as chromosomally integrated HHV-6 DNA (ciHHV-6) individuals. As of late, the importance of cerebrospinal fluid (CSF) HHV-6 DNA being present has been, rightfully, under scrutiny due to the mistaken diagnosis of encephalitis due to the presence of viral DNA in the CSF of ciHHV-6 individuals. Our case represents one of the few individuals with HHV-6 encephalitis in a young immunocompetent male, whose diagnosis was confirmed by quantitative polymerase chain reaction (PCR) and showed complete response to ganciclovir without any further complication.

## Case presentation

A 26-year-old Caucasian male with a past medical history of generalized tonic-clonic seizures was taken by ambulance after his family found him confused and wandering the neighborhood. The patient’s family last saw him in his normal state the night prior to admission, and there was no history of recent travel or new medications. His vaccination status was up-to-date. Of note, the patient had a head-on motor vehicle collision three years ago resulting in monthly seizures despite taking a daily maintenance dose of lamotrigine at 200 milligrams. On arrival, the patient’s temperature was 38.2°C, blood pressure was 94/67 mmHg, heart rate was 108 beats per minute, and saturation SPO2 was 98% on room air. Initial fingerstick blood glucose at bedside was 103 mg/dL. He was alert and oriented only to self and time, with a Glasgow Coma Scale of 11, as well as agitated and uncooperative to history and examination. His appearance was disheveled with dry mucous membranes. Examination was only remarkable for erythematous scratches on his hands bilaterally with a 3-cm superficial healing laceration in the center of his forehead. There was no evidence of generalized or focal neurological deficits, including signs of meningeal inflammation.

Initial labs were remarkable for leukocytosis (24.2 x10^3^/mm^3^) with a left shift and bandemia (segmented neutrophils 80, bands 14), elevated procalcitonin (8.75 ng/mL), acute kidney injury (blood urea nitrogen 24 mg/dL, creatinine 1.76 mg/dL), elevated liver enzymes (AST 271 unit/L, ALT 77 unit/L), and elevated creatinine kinase (14,205 unit/L). A urine drug screen was positive for tetrahydrocannabinol (THC), and a urinalysis demonstrated colorless urine with characteristic myoglobinuria including large gross urine blood with 0-3 red blood cells (Table 1).

**Table 1:** laboratory tests on admission CBC, complete blood count; WBC, white blood cells; CMP, comprehensive metabolic panel; BUN, blood urea nitrogen; CR, creatinine; AST, aspartate aminotransferase; ALT, alanine aminotransferase; CK, creatine phosphokinase; RBCs, red blood cells; CSF, cerebrospinal fluid analysis

Laboratory test	Results	Normal references
CBC		
WBCs (x10^3^ cells/mm^3^)	24.2	4.5-11
Band cell (%)	14	0-10
CMP		
BUN mg/dL	24	6-24
Cr (mg/dL)	1.76	0.74-1.35 in normal adult men
AST (U/L)	271	0-40
ALT (U/L)	77	0-44
CK (U/L)	14205	30-223
Procalcitonin (ng/mL)	8.75	<0.1
Urinalysis test		
Color/appearance	Cloudy yellow	Clear yellow
Gross blood	Large	Negative
RBCs (cells/mm^3^)	0-3	0-3
WBCs (cells/mm^3^)	0-3	0-3
Leukocyte esterase	Negative	Negative
Nitrites	Negative	Negative
Bacteria	Rare	rare
Urine drug test	Positive for Tetrahydrocannabinol (THC)	Negative
CSF		
Specific gravity	1020	1006-1007
Appearance	Clear/colorless	Clear/colorless
Glucose (mg/dL)	67	50-75
Protein (mg/dL)	59	15-45
RBCs (cells/mm^3^)	230	0-5 cells
WBCs (cells/mm^3^)	5 cells/mm^3^	0-5 cells
Gram stain	Negative	Negative
Meningitis/encephalitis panel by polymerase chain reaction test	Positive HHV-6 DNA detected	Negative
Microbial culture	Negative	Negative

Acetaminophen, salicylate, and alcohol levels were all within normal limits. Electrocardiogram (EKG) was remarkable for sinus tachycardia. A computed tomography (CT) without contrast of the brain and cervical spine as well as a frontal chest X-ray were normal. While in the Emergency Department (ED), he was treated with 3 liters of IV fluid boluses for rhabdomyolysis and hemodynamic stability and was given 4 milligrams of oral lorazepam and 5 milligrams of haloperidol for agitation, 10 milligrams of IV dexamethasone out of concern for possible meningitis/encephalitis, and 650 milligrams of acetaminophen for the fever. The patient underwent a complete septic workup including blood cultures, a lumbar puncture at bedside, empiric IV acyclovir, as well as vancomycin and ceftriaxone until CSF analysis returned. CSF analysis revealed a specific gravity of 1020, with colorless, clear appearance, normal glucose (67 mg/dL), elevated protein (59 mg/dL), RBC count of 230 cells/mm^3^, and WBC count of 5 cells/mm^3^. Meningitis/encephalitis panel by PCR was positive for HHV-6 (quantitative PCR of 7200 copies). All other bacteria, viral, and fungal species were negative, including *E. Coli* K1, *H. influenza*, *L. monocytogenes*, *N. meningitidis*, *S. agalactiae*, *S. pneumonia*, Cytomegalovirus, Enterovirus, herpes simplex virus (HSV) 1, HSV2, H. parechovirus, herpes zoster virus (HZV), Epstein-Barr virus, and Cryptococcus. Subsequent labs showed HHV-6 quantitative PCR in plasma of 6,200 copies/mL indicating HHV-6 viremia. A decision was made to transfer the patient to the medical intensive care unit for further management of viral encephalitis. IV ganciclovir 5 mg/kg daily and maintenance fluids with normal saline at 150 cc/hour were added to his regimen. He was maintained on lamotrigine 100 milligrams twice daily, and basic metabolic panels and complete blood count were monitored during his hospital stay. Neurology performed a video electroencephalogram (EEG) study, which was indicative of an epileptogenic lesion in the left temporal area and several electrographic seizures were recorded.

Later, magnetic resonance imaging (MRI) was obtained of the brain, which did not reveal any structural abnormalities or acute enhancement. Two repeated COVID-19 nasopharyngeal swabs for accuracy were negative. Peripheral blood cultures, CSF cultures, and CSF acid-fast bacilli demonstrated no growth during hospitalization nor at eight weeks post-admission. Infectious disease was consulted who ordered HIV and RPR which were non-reactive, in addition to CD4 and CD3 counts, which were also within normal limits. One week later, the patient showed a favorable clinical response in his encephalitis and rhabdomyolysis. He was discharged on lamotrigine 50 milligrams twice daily and oxcarbazepine 150/450 milligrams twice daily, and IV ganciclovir 5 mg/kg daily was switched to PO valganciclovir 450 milligrams, according to his estimated creatinine clearance (CrCl), every morning for another two weeks upon discharge with a plan to repeat peripheral-blood HHV-6 PCR until undetectable. A follow-up lab workup one week after discharge showed high levels of the patient’s HHV-6 IgG antibodies that were undetectable at hospital admission.

## Discussion

HHV-6 is well known for being the cause of roseola infantum (also known as exanthema subitum or sixth disease) as most infections take place in the very young, usually less than three years of age [1]. Many Studies in Japan have shown that primary HHV-6 infection in children could be complicated by neurological sequelae because of excessive cytokine release [2]. These complications include encephalitis, acute necrotizing encephalitis, acute encephalopathy with biphasic seizure syndrome, and/or demyelinating disease [3]. HHV-6 encephalitis can occur in an adult as a primary infection or reactivation, but often it occurs in the immunocompromised, hematopoietic stem cell transplantation (HSCT) patients, and solid organ transplant recipients [4-5]. Our 26-year-old adult patient fell outside of these three categories as he was immunocompetent without a history of HSCT or organ transplantation.

Pathophysiologically, there are three ways HHV-6 can infect a host: primary infection, reactivation of a latent infection, and activation of ciHHV-6. CiHHV-6 is the incorporation of the viral genome into the subtelomeric region of the host’s chromosomes via homologous recombination and can be transmitted to progeny affecting every cell of the offspring’s body [6]. In developed countries, individuals with ciHHV-6 make up approximately 0.2-1% of the population, and knowing if a patient is affected may significantly influence medical management and treatment [6]. As of late, the importance of HHV-6 DNA being present in CSF has been, justifiably, under scrutiny [1]. This is due to the discovery of viral DNA in the CSF of ciHHV-6 affected individuals and mistakenly attributing the diagnosis to a primary, actively replicating infection [1].

In order to deviate from misdiagnosis, many testing modalities have been used to assist in the diagnosis of HHV-6 infection, but quantitative PCR, with its high sensitivity and specificity, has become the gold standard in detecting and quantifying HHV-6 DNA [6-7]. Other methods, such as serology, cell culture, and antigen detection, can play an important role in the diagnosis of HHV-6 infection as well [6]. When diagnosing an active infection in a specific body compartment, as in our case of encephalitis, PCR should be used to compare the viral load in whole blood as well as the specific body compartment [6]. Whole blood PCR helps differentiate ciHHV-6 from an actively replicating infection since the viral load routinely exceeds 1 million copies per mL in ciHHV-6 affected individuals [7]. Unfortunately, whole blood PCR was not tested in our patient; however, studies state that for local and active CNS infections, only detectable levels of HHV-6 DNA in the CSF are needed for diagnosis regardless of the level of viral DNA in a whole blood sample [6,8,9].

Unlike CSF, the increased viral load in plasma is not a definitive diagnosis of active HHV-6 infection. This patient had 6,200 copies/mL. Patients affected by ciHHV-6 can have increased copies of viral DNA in the plasma due to viral genome release from cellular degradation in preparatory laboratory processing [6-10]. It is possible that viral DNA could have been preserved in the plasma due to cell lysis while undergoing either the centrifugation process or with abnormal storage temperature prior to processing [11]. Even with this possibility, there is incomplete, but robust, evidence against this patient having ciHHV-6 due to the increased viral load in the CSF without an accompanying rise in nucleated cell count. Compared to a ciHHV-6 patient, an increased number of nucleated cells in the CSF would be expected in order to elevate the number of viral DNA copies [1,11]. In addition, serology showed the patient’s HHV-6 IgG antibodies were undetectable at hospital admission but subsequently rose to a high level over the accompanying weeks indicating an active infection was present. Interestingly, a prior study demonstrated a seemingly paradoxical decrease in antibody levels in individuals with ciHHV-6, not an increase [11].

Reactivation of a latent infection is another avenue toward HHV-6 replication and encephalitis but carries its own hurdles for diagnosis. Our current understanding of the reactivation mechanism is limited; however, it seems to be precipitated by discrete viral gene expression followed by an inflammatory response rather than a modest level of replication, as seen in this case [6]. It is not fully understood in what state the virus exists within cells. There may be a consistent, low-level production or simply maintenance of the genome, but experiments have shown that HHV-6 can exist in an episomal state within the nucleus of some cervical cancer cell lines. In addition, the expression of the viral U-94 and immediate early (IE) genes have been associated with latent infection and should be detectable if latency is the cause [6]. Genetic testing of the U-94 or IE viral genes were not instituted for this patient; however, the other aspects of this case make it unique and support our conclusion that active replication of HHV-6 was the cause of our patient’s condition.

There are a few limitations to our report. The first is the lack of a whole blood PCR test. Without it, we cannot definitively rule out the possibility that this patient is part of the 0.2-1% population affected by ciHHV-6 [6]. Considering this, it may be possible that an undetected inflammation caused his altered mental state and was suppressed during treatment with dexamethasone. Another possibility would be an undetected virus responded to the antiviral treatment, even though all other likely causative agents were accounted for. Lastly, the lack of U-94 and IE viral genetic testing cannot rule out the reactivation of a latent infection.

Even though emphasis has been placed on careful diagnosis to avoid attributing encephalitis to a ciHHV-6 individual, clinical symptoms, exclusion of other causes, and the end results of treatment must also be considered [5]. In reference to this patient’s symptoms, the clinical presentation of encephalitis with uncontrolled temporal lobe epilepsy (TLE) increases the possibility of active HHV-6 replication. These studies have suggested that astrocytes are susceptible to infection by HHV-6, causing their mechanism of glutamate uptake to be altered, thus impairing the neural excitatory balance seen in epilepsy [12]. This concept explains how HHV-6 could be the cause of one-third of febrile status epilepticus cases [13]. Furthermore, it explores HHV-6’s association with TLE, a common form of epilepsy, with or without a history of HHV-6 encephalitis [12]. Clinically, the refractory manifestation of TLE has been associated with previous neurological damage such as febrile seizures, tumor, head trauma, or even infections. However, according to a recent comprehensive meta-analysis of the correlation between HHV-6 and refractory TLE, it was demonstrated that HHV-6 was found more frequently in TLE-based temporal lobe resections than in tissue from the control group [14]. Given that our patient had a history of uncontrolled seizure activity in his left temporal lobe despite being on maintenance anti-epileptic medication and with recorded exacerbation during his hospitalization, it is conceivable that HHV-6 was the underlying and contributing factor in causing his seizure, particularly after his car accident.

Also, an increased CSF nucleated cell count would be expected if another viral, bacterial, or fungal organism was present [15-17]. In this case, WBC count remained within the normal range, which argues against the presence of ciHHV-6 or any other causative organism. Additionally, the positive toxicology assay for THC would not explain such a profound delirium coinciding with the other physical and laboratory findings. Furthermore, the temporal relationship between the onset of symptoms followed by a full recovery in response to treatment with ganciclovir and valganciclovir in the absence of other causative agents is far too serendipitous to be happenstance [6]. Because of these reasons, it should be deduced that a replicating HHV-6 viral infection is the culprit of our patient’s condition.

## Conclusions

As HHV-6 has been frequently shown to be the etiology in contributing to the cause of encephalitis or meningoencephalitis in immunocompromised patients, there have been very few known cases, including our patient, where HHV-6 has been implicated in causing encephalitis in immunocompetent adults. Studies have emphasized the care that must be taken in the interpretation of laboratory results to avoid attributing encephalitis to ciHHV-6 individuals. Undoubtedly, additional studies are required to investigate the clinical scenario in which an immunocompetent patient presenting with encephalopathy should be tested for HHV-6 as a potential etiology. In those patients found to have a confirmed diagnosis of HHV-6 encephalitis, strong consideration for the use of antiviral therapy should be given as a crucial part of the treatment management.
